# Empirical prescribing of penicillin G/V reduces risk of readmission of hospitalized patients with community-acquired pneumonia in Norway: a retrospective observational study

**DOI:** 10.1186/s12890-020-01188-6

**Published:** 2020-06-15

**Authors:** June Utnes Høgli, Beate Hennie Garcia, Kristian Svendsen, Vegard Skogen, Lars Småbrekke

**Affiliations:** 1 Regional Centre for Infection Control, University Hospital of North Norway, N-9038 Tromsø, Norway; 2 Department of Pharmacy, Faculty of Health Sciences, UiT – The Arctic University of Norway, N-9037 Tromsø, Norway; 3 Hospital Pharmacy of North Norway Trust, N-9291 Tromsø, Norway; 4 Department of Infectious Diseases, Division of Internal Medicine, University Hospital of North Norway, N-9038 Tromsø, Norway; 5 Department of Clinical Medicine, Faculty of Health Sciences, UiT – The Arctic University of Norway, N-9037 Tromsø, Norway; 6 Infectious Diseases Unit, LaFe University Hospital, Valencia, Spain

**Keywords:** Community-acquired pneumonia, Antibiotics, Guideline, Clinical outcome, Norway, Antibiotic stewardship program

## Abstract

**Background:**

Norwegian guideline recommendations on first-line empirical antibiotic prescribing in hospitalised patients with community-acquired pneumonia (CAP) are penicillin G/V in monotherapy, or penicillin G in combination with gentamicin (or cefotaxime) in severely ill patients. The aim of this study was to explore how different empirical antibiotic treatments impact on length of hospital stay (LOS) and 30-day hospital readmission. A secondary aim was to describe median intravenous- and total treatment duration.

**Methods:**

We included CAP patients (≥18 years age) hospitalised in North Norway during 2010 and 2012 in a retrospective study. Patients with negative chest x-ray, malignancies or immunosuppression or frequent readmissions were excluded. We collected data on patient characteristics, empirical antibiotic prescribing, treatment duration and clinical outcomes from electronic patient records and the hospital administrative system. We used directed acyclic graphs for statistical model selection, and analysed data with mulitvariable logistic and linear regression.

**Results:**

We included 651 patients. Median age was 77 years [IQR; 64–84] and 46.5% were female. Median LOS was 4 days [IQR; 3–6], 30-day readmission rate was 14.4% and 30-day mortality rate was 6.9%. Penicillin G/V were empirically prescribed in monotherapy in 51.5% of patients, penicillin G and gentamicin in combination in 22.9% and other antibiotics in 25.6% of patients. Prescribing other antibiotics than penicillin G/V monotherapy was associated with increased risk of readmission [OR 1.9, 95% CI; 1.08–3.42]. Empirical antibiotic prescribing was not associated with LOS. Median intravenous- and total treatment duration was 3.0 [IQR; 2–5] and 11.0 [IQR; 9.8–13] days.

**Conclusions:**

Our findings show that empirical prescribing with penicillin G/V in monotherapy in hospitalised non-severe CAP-patients, without complicating factors such as malignancy, immunosuppression and frequent readmission, is associated with lower risk of 30-day readmission compared to other antibiotic treatments. Median total treatment duration exceeds treatment recommendations.

## Background

Community-Acquired Pneumonia (CAP) is the leading cause of death due to infectious diseases in adults worldwide. The annual adult incidence range from 1 to 8 per 1000 inhabitants, is higher in men and increases with age. The 30-day hospital readmission rate range from 15 to 20% [1–5]. Reported 30-day mortality rate due to CAP in Scandinavia ranges from 7 to 11% [3, 6].

*Streptococcus pneumoniae* is the most frequent identified cause of CAP. Other common pathogens include *Haemophilus influenzae*, *Mycoplasma pneumoniae* and respiratory viruses [3, 7–9]. Obtaining a microbiological diagnosis is difficult, and an aetiological diagnosis in CAP is unconfirmed in up to 50% of patients [7–9]. In Norway, < 1% of *S.pneumoniae* blood culture and respiratory isolates are resistant for penicillin G/V, and 6 and 8.2% of *S.pneumoniae* in blood culture- and respiratory isolates are resistant to erythromycin, respectively [10]. For *H.influenzae* blood culture isolates the prevalence of beta-lactamase and chromosomal resistance are 17.8 and 16.1%, respectively [10].

Appropriate treatment for CAP is reflected by recommendations in clinical practice guidelines (CPGs). Geographic location and host factors predict the causative pathogen and antimicrobial resistance (AMR). Consequently, recommendations in CPGs can differ between countries. In most European and American guidelines a β-lactam (type of recommended β-lactam differs between countries) combined with a macrolide, or a respiratory fluoroquinolone in monotherapy, is recommended as empirical treatment for hospitalised CAP-patients [11–13]. Scandinavian and Dutch guidelines recommends narrow spectrum penicillin G/V (or ampicillin) in monotherapy as first-line empirical treatment in non-severe CAP with no routinely empirical coverage for atypical pathogens [14–17]. Recommendations for severely ill CAP patients varies, and the Norwegian guideline recommends penicillin G in combination with gentamicin or cefotaxime in monotherapy for patients where atypical pathogens are not suspected [15].

Appropriate antibiotic prescribing is essential for patient safety and outcome, and for reducing emergence of AMR [18]. A Danish study recently found no association between empirical treatment with penicillin G/V and mortality in mild to moderate CAP [3]. Inappropriate prolonged treatment has been associated with longer LOS, higher costs and an increase in adverse drug reactions without altering treatment effect, number of recurrent infections and mortality [19, 20].

The aim of this study was to explore how different empirical antibiotic treatments impact on LOS and 30-day hospital readmission. In addition, we aimed to describe median intravenous (IV) and total treatment duration.

## Methods

### Setting and study population

The University hospital of North Norway (UNN) is a 500-bed hospital in the North Norway health region. UNN serves about 195,000 inhabitants and is divided into three subunits located in three different towns; Harstad, Narvik and Tromsø.

**Fig. 1:**
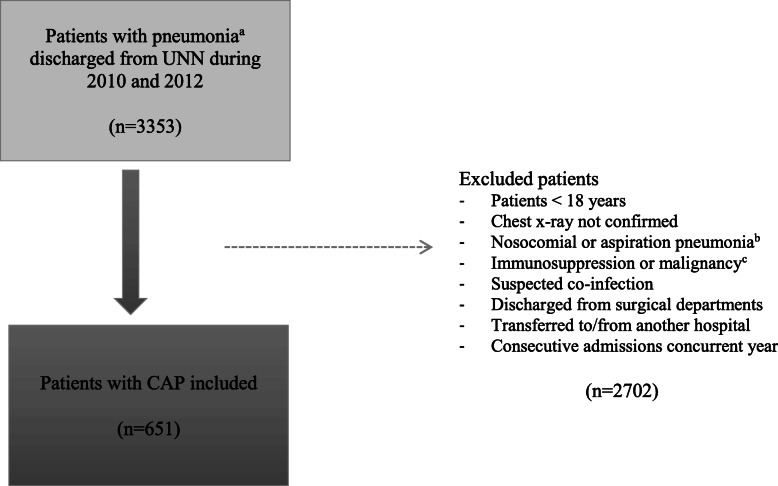
Patient inclusion process. UNN; University Hospital of North Norway. ^a)^ International Statistical Classification of Diseases and Related Health Problems (ICD) 10 codes applied: Pneumonia due to J13; *Streptococcus pneumoniae*, J14; *Hemophilus influenzae*, J15.0-J15.6; *Klebsiella pneumoniae*, *Pseudomonas*, *Staphylococcus*, other *Stretptococci, Escherchia coli* or other Gram-negative bacteria, J15.7; *Mycoplasma pneumoniae*, J15.8; other specified bacteria, J15.9; Unspecified bacterial pneumonia, J16; *Chlamydia pneumonia* and other specified organism, J18; Bronchopneumoniae, unspecified organism. ^b)^ Nosocomial pneumonia; pneumonia presenting 48 h after admission to hospital. Aspiration pneumonia; pneumonia due to inhalation of either oropharyngeal or gastric contents into the lower airways. Documented in patient notes. ^c)^ Immunsuppresion or maliganancy; Transplanted, cancer, receiving cytostatic drugs, human immunodeficiency virus and immunodeficiency with antibody defects

We conducted a retrospective observational study including patients ≥18 years discharged from UNN during 2010 and 2012 with a CAP diagnosis registered at discharge (ICD-10 codes J13–16 or J18). We excluded patients with no confirmed chest x-ray, nosocomial or aspiration pneumonia, immunosuppression or malignancies, (suspected) co-infection, discharged from surgical departments, transferred from or to other hospitals and with consecutive admissions due to CAP the current year (patients could only be included once per year in the study), see Fig. 1.

### Data collection and clinical definitions

We retrospectively extracted the following patient data from electronic records, medication charts and laboratory data; age, gender, antibiotics used pre-hospitalisation (yes/no), nursing home residency status, penicillin allergy status, relevant comorbidities (chronic obstructive pulmonary disease (COPD), heart failure and diabetes mellitus I or II), infection-relevant clinical and laboratory data from the first 3 days of hospitalisation (blood pressure, heart rate, respiratory rate, body temperature, oxygen saturation, leucocytes and c-reactive protein), microbiological tests ordered and pathogens identified. We calculated severity according to CRB-65 (confusion, respiratory rate, blood pressure, age ≥ 65 years) based on information at admission [21].

We collected the complete antibiotic medication list for the hospital stay (drug, dose, route of administration and duration), including any amendment of empirical antibiotic prescribing within first 3 days (i.e. switch to a broader spectrum IV antibiotic, or addition of a new antibiotic to the current regime). We defined total treatment duration as length of antibiotic treatment in hospital plus the prescribed length of treatment at discharge. Antibiotic treatment started pre-hospitalisation was not included in calculation of treatment duration. The clinical outcome measures LOS, all-cause 30-day readmission (unplanned, calculated from time of discharge) and all-cause 30-day mortality were collected from the hospital administrative system.

Patients were categorized into three groups according to type of empirical antibiotic prescribed:1) penicillin G/V in monotherapy, 2) penicillin G in combination with gentamicin and 3) all other antibiotic treatments.

### Statistics

We used Microsoft® Office Excel 2010 and STATA®14 for data analysis. Descriptive statistics are reported as counts and percentages for categorical data, and median and 25th to 75th interquartile range for continuous data. We performed descriptive statistics on the crude study population and a stratified analysis on patient characteristics and clinical outcomes for the three different empirical antibiotic treatment categories. In the stratified analysis/group comparison, we tested difference in proportions (categorical data) with Pearson’s χ2-test and difference in means (continuous data) with ANOVA test. A *P*-value of < 0.05 was considered statistically significant.

We used multivariable linear regression to explore the impact of empirical antibiotic prescribing (exposure) on LOS (outcome), and logistic regression to explore the impact of empirical antibiotic prescribing (exposure) on 30-day readmission (outcome).

Directed Acyclic Graphs (DAGs) were applied for statistical model selection [22]. The DAGs were created and analysed in the browser-based program DAGitty version 2.0. (http://www.dagitty.net), see Additional file 1. We adjusted for the following confounders; CRB-65, comorbidities, age, gender, pathogen, year of admittance, hospital and nursing home residence. LOS, 30-day readmission, IV- and total treatment duration were recorded as missing for patients who died in hospital. Only patients with complete data on outcome and confounders were included in the regression analyses.

## Results

### Study population and outcomes

We included 651 (19.4%) out of the 3342 patients that were discharged with ICD-10 code J13–16 or J18. Median age was 77 years (IQR; 64–84] and 46.5% were female. An aetiological agent was identified in 21% of patients, and *S.pneumoniae* was the most common pathogen detected. About 11% of patients were allergic to penicillin. Median LOS was 4 days [IQR; 3–6], and the 30-day readmission rate was 14.4%. The 30-day mortality rate was 6.9%. We calculated that 7.5% of patients had high risk of mortality according to CRB-65 (score ≥ 3). See Table 1.

**Table 1:** Patient characteristics (*n* = 651)

Patient characteristics	Total	
	n	(%)
Gender, female	303	(46.5)
Age, years, median (IQR)	77	(64-84)
Nursing home residents	70	(10.8)
Penicillin allergy^a^	70	(10.8)
Comorbidities		
COPD	205	(31.5)
Heart failure	140	(21.5)
Diabetes mellitus I or II	85	(13.1)
CRB-65 score^b^		
0	110	(16.9)
1	225	(34.9)
2	179	(27.5)
3	43	(6.6)
4	6	(0.9)
Missing data	88	(13.5)
Registered in admission notes	1	(0.2)
Antibiotic use pre-hospitalization	171	(26.3)
Empirical antibiotic prescribing		
Penicillin G/V monotherapy	335	(51.5)
Penicillin G + gentamicin	149	(22.9)
Other antibiotics	167	(25.7)
Treatment duration		
IV treatment duration, median (IQR)	3	(2-5)
Total treatment duration, median (IQR)	11	(9.8-13)
Microbiological diagnostics		
Blood culture	499	(76.7)
Nasopharynx	179	(27.5)
Expectorate	100	(15.4)
Pneumococcal urinary antigen test	327	(50.2)
Legionella urinary antigen test	51	(7.8)
Serology *M. or C.pnuemoniae*	19	(2.9)
Other	220	(33.8)
None	68	(10.4)
Aetiology		
*S.pneumoniae*	61	(9.4)
*M. or C. pneumoniae*	22	(3.4)
*H.influenzae*	11	(1.7)
*S.aureus*	7	(1.1)
Other bacteria	14	(2.2)
Influenza virus A or B	12	(1.8)
Other respiratory viruses	18	(2.8)
None identified	514	(79.0)
Clinical outcomes		
30-day readmission	90	(14.4)
Length of stay in hospital, median	4	(3-6)
30-day mortality	44	(6.9)

*CRB-65* Confusion, respiration, blood pressure and age ≥ 65y, *COPD* Chronic obstructive pulmonary disease, *IQR* inter quartile range, *IV* intravenous

^a^Documented penicillin allergy in patient notes to beta-lactams

^b^Scoring made retrospectively based on admission data/journal data

### Antibiotic prescribing

Penicillin G/V were empirically prescribed in monotherapy in 51.5% of patients, penicillin G and gentamicin in combination in 22.9% and other antibiotics in 25.6% of patients. Cefotaxime, doxycycline and erythromycin were the most commonly prescribed antibiotics among those receiving other antibiotics. See Additional file 2 for a description of all empirical antibiotics prescribed and Additional file 3 for a description of choice of empirical antibiotic versus subsequent bacterial pathogen identified. Empirical antibiotic prescribing was amended before day three in 16.3% of the patients. Median IV treatment duration was 3.0 days (mean 3.7) and median total treatment duration was 11.0 days (mean 11.6).

### Group comparison of three different empirical treatments

Stratified results on patient characteristics and clinical outcomes for the different empirical antibiotic treatments are given in Table 2. The results show that patients prescribed ‘other antibiotic treatments’ had higher 30-days readmittance rate, had higher prevalence of COPD and penicillin allergy, and were more frequently prescribed antibiotics pre-hospitalisation. In addition, we observed differences in nursing home residency status and IV- and total treatment duration between the treatments. Patients prescribed other antibiotics did not have more severe CAP, measured by CRB-65, than patients prescribed penicillin G/V.

**Table 2:** Stratified results for different empirical prescribing. Unadjusted analysis

	Monotherapy penicillin G/V (*n* = 335)	Combination penicillin G and gentamicin (*n* = 149)	Other antibiotics (*n* = 167)	*p*-value*
	n (%)	n (%)	n (%)	
Age, years				
Mean	72.8	70.8	70.4	0.29
Gender, female	150 (44.8)	67 (45.0)	86 (51.5)	0.33
Comorbidities				
COPD	94 (28.1)	44 (29.5)	67 (40.1)	0.02
Heart failure	73 (21.8)	26 (17.5)	41 (24.6)	0.30
Diabetes mellitus I or II	50 (14.9)	13 (8.7)	22 (13.2)	0.17
Nursing home resident	27 (8.1)	22 (14.8)	21 (12.6)	0.06
Penicillin allergy	8 (2.4)	2 (1.3)	60 (35.9)	0.00
CRB-65				0.11
0	57 (17.0)	23 (15.4)	30 (18.0)	
1	127 (37.9)	38 (25.5)	60 (35.9)	
2	87 (26.0)	46 (30.9)	46 (27.5)	
3	15 (4.5)	17 (11.4)	11 (6.6)	
4	2 (0.6)	2 (1.3)	2 (1.2)	
Missing data	47 (14.0)	23 (15.4)	18 (10.8)	
Antibiotic use pre-hospitalization	64 (19.1)	40 (26.9)	67 (40.1)	0.00
Treatment amended within 3 days	56 (16.7)	25 (16.8)	25 (15.0)	0.87
IV treatment duration, mean (days)	3.4	4.1	4.1	0.01
Total treatment duration, mean (days)	11.4	12.3	11.3	0.04
Clinical outcomes				
30-day readmission	38 (11.6)	19 (13.4)	33 (20.8)	0.02
Length of stay (mean)	5.1	5.8	5.0	0.20
30-day mortality	20 (6.0)	11 (7.4)	13 (7.8)	0.70

*CRB-65* Confusion, respiration, blood pressure and age ≥ 65y, *COPD* Chronic obstructive pulmonary disease, *IV* intravenous

*Categorical data analyzed using Pearson’s χ2-test and continuous data using ANOVA test

### Association with length of stay in hospital (*n* = 626)

Neither empirical prescribing of penicillin G/V in monotherapy, penicillin G in combination with gentamicin nor other antibiotic treatments were associated with LOS. A 1 year increase in age increased mean LOS with an average of 0.04 days [95% CI; 0.02, 0.07]. Heart failure was associated with an increase of 1.4 days in mean LOS [95% CI; 0.6, 2.2], and a CRB-65 score of 3 was associated with an increase of 1.9 days in mean LOS [95% CI; 0.4, 3.50]. Positive findings of *S.pneumoniae*, *S.aureus* and other bacteria was associated with 1.22 [95% CI; 0.10, 2.34], 4.10 [95% CI; 1.24, 6.96] and 5.19 [95% CI; 2.98, 7.40] days longer mean LOS, respectively. Admission to hospital in 2012 compared to 2010 was associated with − 0.42 [95% CI; − 0.73,-0.11] shorter mean LOS. We also observed differences between hospitals. See Table 3 for the complete table with adjusted coefficients.

**Table 3:** Multivariable regression analysis. Association between empirical antibiotic prescribing, various covariates and length of hospital stay (LOS) (*n* = 626) and 30-day readmission (*n* = 609), respectively

Variable	Length of hospital stay^a^		30-day readmission^b^	
	Adjusted coefficient	[95% CI]	Adjusted odds ratio	[95% CI]
Empirical antibiotic prescribing				
Penicillin G/V in monotherapy	0	Ref.	1	Ref.
Penicillin G + gentamicin	0.68	[−0.10, 1.46]	1.39	[0.73, 2.66]
Other antibiotics	0.33	[−0.43, 1.09]	1.92	[1.08, 3.42]
Antibiotic use pre-hospitalization	−0.25	[− 0.95, 0.46]	1.15	[0.66, 1.99]
Female	0.08	[−0.53, 0.69]	0.50	[0.30, 0.82]
Age	0.04	[0.02, 0.07]	1.01	[0.99, 1.03]
Comorbidities				
COPD	0.22	[−0.46, 0.86]	2.07	[1.26, 3.41]
Heart failure	1.40	[0.62, 2.17]	1.45	[0.83, 2.51]
Diabetes Mellitus I or II	0.38	[−0.52, 1.27]	1.22	[0.63, 2.37]
Nursing home resident	−0.98	[−2.05,2.17]	1.40	[0.64, 3.07]
Year admitted (2012 vs. 2010)	−0.42	[−0.73,-0.11]	0.85	[0.66, 1.08]
Hospital				
Hospital A	0	Ref	1	Ref.
Hospital B	0.81	[−0,14, 1.76]	0.74	[0.37, 1.50]
Hospital C	1.10	[0.34, 1.87]	0.51	[0.29, 0.88]
Pathogens				
None identified	0	Ref.	1	Ref.
*S.pneumoniae*	1.22	[0.10, 2.34]	0.96	[0.37, 2.47]
*H.influenzae*	2.13	[− 041, 4.67]	3.01	[0.65, 14.02]
*M. or C. pneumoniae*	0.75	[−0.98, 2.48]	0.39	[0.05, 3.22]
*S.aureus*	4.10	[1.24, 6.96]	5.24	[0.99, 27.6]
Other bacteria	5.19	[2.98, 7.40]	1.38	[0.26, 7.38]
Influenza virus A or B	1.86	[−0.83, 4.55]	–	–
Other respiratory viruses	0.44	[−1.56, 2.44]	0.85	[0.10, 6.98]
Two or more pathogens	1.40	[−1.30, 4.10]	–	–
CRB-65 score				
0	0	Ref.	1	Ref.
1	−0.19	[− 1.24, 0.85]	0.82	[0.32, 2.06]
2	0.50	[−0.65, 1.64]	0.99	[0.37, 2.61]
3	1.92	[0.35, 3.50]	0.71	[0.19, 2.66]
4	1.63	[−2.85, 6.11]	–	–
Missing data	−0.27	[−1.55, 1.01]	0.78	[0.26, 2.39]

*CI* confidence interval, *COPD* Chronic obstructive pulmonary disease, *CRB-65* Confusion, Respiration, Blood pressure and Age

^a^ Linear regression to explore impact of empirical antibiotic prescribing on length of hospital stay. Adjusted for antibiotic use pre-hospitalization, gender, age, comorbidities, nursing home resident, year admitted (2012 vs. 2010), hospital, pathogens and severity of infection (CRB-65)

^b^ Logistic regression to explore impact of empirical antibiotic prescribing on 30-day readmission. Adjusted for antibiotic use pre-hospitalization, gender, age, comorbidities, nursing home resident, year admitted (2012 vs. 2010), hospital, pathogens and severity of infection (CRB-65)

### Association with 30-day readmission (*n* = 609)

OR for 30-day readmission for prescribing ‘other antibiotics’ compared with penicillin G/V in monotherapy (reference) was 1.9 [95% CI; 1.08–3.42]. OR for prescribing penicillin G in combination with gentamicin compared with penicillin G/V in monotherapy (reference) was 1.4 [95% CI; 0.73–2.66].

Female patients had lower risk of 30-days hospital readmission [OR 0.5, 95% CI; 0.30–0.82], while patients with COPD had higher risk of readmission [OR 2.07, 95% CI; 1.26–3.41]. In addition, we found variations in risk of 30-day readmission between the three hospitals. See Table 3 for the complete table with adjusted odds ratio.

## Discussion

In this setting with low levels of AMR among common airway pathogens and extensive use of penicillin G/V, we found that empirical treatment with penicillin G/V in monotherapy was associated with reduced risk of 30-day readmission compared to other empirical antibiotic treatments. The 30-day mortality rate was 6.9%, and the median IV and total treatment duration was 3.0  (mean 3.7) and 11.0 days (mean 11.6), respectively.

The extensive use of penicillin G/V in Scandinavian countries is in contrast to other countries where level of penicillin resistance limits the use of penicillin G/V [20]. Whether this approach is associated with favourable clinical outcomes has been sparsely documented until recently. A Danish study found no association between penicillin G/V in monotherapy for non-severe CAP in respect of mortality [3]. As far as we know, association between empirical prescribing with penicillin G/V and risk of readmission has for similar CAP-cohorts not been investigated. Altogether, our findings suggest that prescribing penicillin G/V for non-severe CAP is sound and safe with regard to desired patient outcome (LOS, mortality and readmission). With increasing concerns about AMR and focus on the importance of appropriate antibiotic prescribing, the treatment traditions in Scandinavia and the Netherlands illustrates that it is possible to use narrow spectrum treatments in a setting with low level of AMR.

For severe CAP, the Norwegian CPG recommends penicillin G in combination with gentamicin or cefotaxime in monotherapy. The latter treatment option is also seen in Dutch guidelines for patients with CURB-65 3–5 in non-ICU-settings (i.e. second or third generation cephalosporines) [14]. The evidence for recommending gentamicin is scarce, but gentamicin in combination with penicillin G has been used for severe CAP for decades in Norway [23]. Using gentamicin prevents excessive use of cephalosporines [14, 23]. The combination therapy covers the main expected pathogens in severe-CAP; gentamicin is primarily efficient in case of bacteraemia covering potential gram-negative pathogens [10]. Penicillin G covers *S.pnuemoniae* and *H.influenzae* (in high doses and in absence of resistance) [10].

An aetiological agent (including both viral and bacterial pathogens) was identified in 21% of patients in our study. This is in agreement with Danish findings by Egelund et al. [3], but low compared to two recent Norwegian studies by Holter et al. [9] and Roysted et al. [8]. These authors found an aetiological agent in 63 and 37% of CAP-patients. *S.pneumoniae* was the most prevalent bacterial pathogen both in our study and in the studies by Egelund et al., Holter et al. and Roysted et al. with 9, 5, 30 and 20%, *H.influenzae* was identified in 2, 4, 5 and 6% and *M.pneumoniae* or *C.pneumoniae* in 3, 3, 6 and 3%, respectively [3, 8, 9]. While *Legionella species* was not identified in our study, the Danish study by Egelund et al. identified *Legionella species* in < 1%. The two Norwegian studies by Holter et al. and Roysted et al. identified *Legionella species* in 3 and 6%, respectively [8, 9]. Holter et al. describes that nearly all *Legionella*-cases was infected abroad and Roysted et al. describes that eight of the 21 patients diagnosed with *Legionella species* was identified by serology post-discharge and these patients recovered without specific *Legionella* treatment. In addition, some of the patients included in the study by Roysted et al. may be part of a local outbreak in 2008 [24]. Overall, only 40–70 cases have been diagnosed annually with *Legionella species* in Norway the last 5 years and more than half of the patients are infected abroad [10].

The Norwegian CPG do not recommend empiric antibiotic treatment for atypical pathogens if atypical pathogens are not clinically suspected. This specific recommendation lean on the low incidence of these pathogens and literature that do not show benefit of survival or clinical efficacy for atypical coverage [15]. From our data we have no indication that not covering empirically for atypical pathogens has negative implications in the overall non-severe CAP population.

Studies investigating associations between empirical antibiotic prescribing and clinical outcomes have primarily focused on mortality as outcome measure [25]. Unfortunately, we had too few patients for a conclusive assessment of association with mortality. Still, our data suggest no negative effect on mortality in this patient selection as 30-day mortality in our study (6.9%) are lower or comparable to other findings from Scandinavia [3, 6].

It seems incomprehensible that prescribing antibiotics with broader spectrum should result in more readmission compared to prescribing penicillin G/V in monotherapy. However, after adjusting for relevant covariates, we are still unable to pinpoint the exact reasons for these findings. Whether it has to do with our outcome measure “all-cause readmissions” and not “pneumonia-specific readmissions” is uncertain, and can unfortunately not be explored as this data has not been collected. However, the advantage of reporting all-cause readmission is that bias inherent with defining exact cause of readmission is avoided [26]. Consequently, applying “all-cause readmission” depends solely on the number of readmissions identified and might therefore be more reliable [26]. Furthermore, the 30-day readmission rate in our study of 14.4% is comparable to findings in other studies [4, 5].

In an observational study it is challenging to attribute causality to an observed association. The selection of statistical model is critical for minimizing bias in estimates when testing association between exposure and outcome. A strength in our study is that we have applied DAGs to structurally approach the minimal set of covariates to include in the model, and we thereby increase statistical efficiency. In addition, applying DAGs to guide assumptions for the regression models increase transparency. Possibly, by collecting more information on co-morbidities, we could further reduce bias in our models. CRB-65, an indicator of severity, does not significantly differ between the groups prescribed penicillin G/V and other antibiotics. Still, due to the retrospective design we cannot rule out selection bias and confounding by indication. Consequently, we cannot rule out that patients treated with penicillin G/V had less severe CAP compared to patients prescribed other antibiotics.

We have identified several areas with room for improvement. One of them is the high level of recorded penicillin allergy (10.8%) which is not in accordance with the estimated prevalence of < 1% [27]. Penicillin allergy testing should be standard care in hospitals, and is increasingly integrated in antibiotic stewardship programs globally [28]. Hospitals that have implemented de-labelling activities have had success in reducing prescription of restricted antibiotics, and it is proven to be safe for the patients [29, 30].

Median IV treatment duration was 3.0 days, which may be considered adequate. However, we suspect that IV treatment duration could have been shorter because our study population mainly comprised non-severe CAP-patients.

A total of 10–14 days antibiotic treatment duration seems to have gained wide acceptance [31, 32], and this is in line with our findings. This is significantly longer than the CPG-recommended duration of 5–7 and 7–10 days in non-severe and severe CAP-patients, respectively. In addition, recent literature indicates that duration as short as 3 days is non-inferior to longer treatment [33].

Our study has several methodological strengths and limitations. First, we have a homogenous study population and consequently a relative precise estimate of the clinical outcomes in the selected population. Second, this is a retrospective observational study and there will always be a risk of bias due to unmeasured variables. Third, the scarcity in patient records of data on infection relevant clinical- and laboratory data from the first 3 days of the hospital stay refrained assessment of time to clinical stability. Fourth, CRB-65 is recommended as a scoring tool for severity in Norway [15]. Surprisingly, we observed that CRB-65 score was documented in only one patient record. Consequently, we had to calculate CRB-scores based on information in admission notes. Our classification of severity of disease may be in conflict with physicians’ judgment at time of empirical prescribing. Still, the distribution of CRB-65 scores is comparable to other studies, with a substantial proportion of patients with low risk of mortality [34]. Fifth, if we had collected data on ICU-admissions, our assumptions on severity could have been strengthened. On the other hand, using ICU-admission as a surrogate for severity is not unproblematic. The decision to admit a patient to an ICU can be due to other considerations than severity and can vary widely between hospitals [35].

## Conclusion

In a Norwegian hospital setting predominated by non-severe CAP patients, we found that prescribing penicillin G/V was associated with lower risk of 30-day readmission compared to other antibiotic treatments. Our results support the national guideline recommendations for empirical antibiotic prescribing for patients presenting without complicating factors such as immunosuppression and frequent readmissions. Our data can be used to reassure clinicians that this treatment is appropriate in this specific setting and patient population.

The proportion of patients with penicillin allergy was high and median total treatment duration unnecessary long.

## Supplementary information

**Additional file 1.** Directed Acyclic Graphs; Association between empirical prescribing (exposure) and readmission or length of stay in hospital (outcomes).

**Additional file 2.** Description of all empirical antibiotics prescribed

**Additional file 3.** Description of empirical antibiotic prescribed versus subsequent bacterial pathogen identified

## Data Availability

The datasets generated and/or analysed during the current study are not publicly available, but are available from the corresponding author on reasonable request.

## Abbreviations

AMR: Antimicrobial resistance
CAP: Community-acquired pneumonia
CPG: Clinical practice guideline
COPD: Chronic obstructive pulmonary disease
CRB-65: Confusion, respiratory rate, blood pressure, age ≥ 65 years
DAGs: Directed Acyclic Graphs
IV: Intravenous
LOS: Length of stay in hospital
UNN: University Hospital of NorthNorway
