# Supplementary figures and images for: Empirical prescribing of penicillin G/V reduces risk of readmission of hospitalized patients with community-acquired pneumonia in Norway: a retrospective observational study
Source: BMC Pulm Med. 2020 Jun 15;20:169. doi: 10.1186/s12890-020-01188-6 (PMC7294665; doi:10.1186/s12890-020-01188-6)

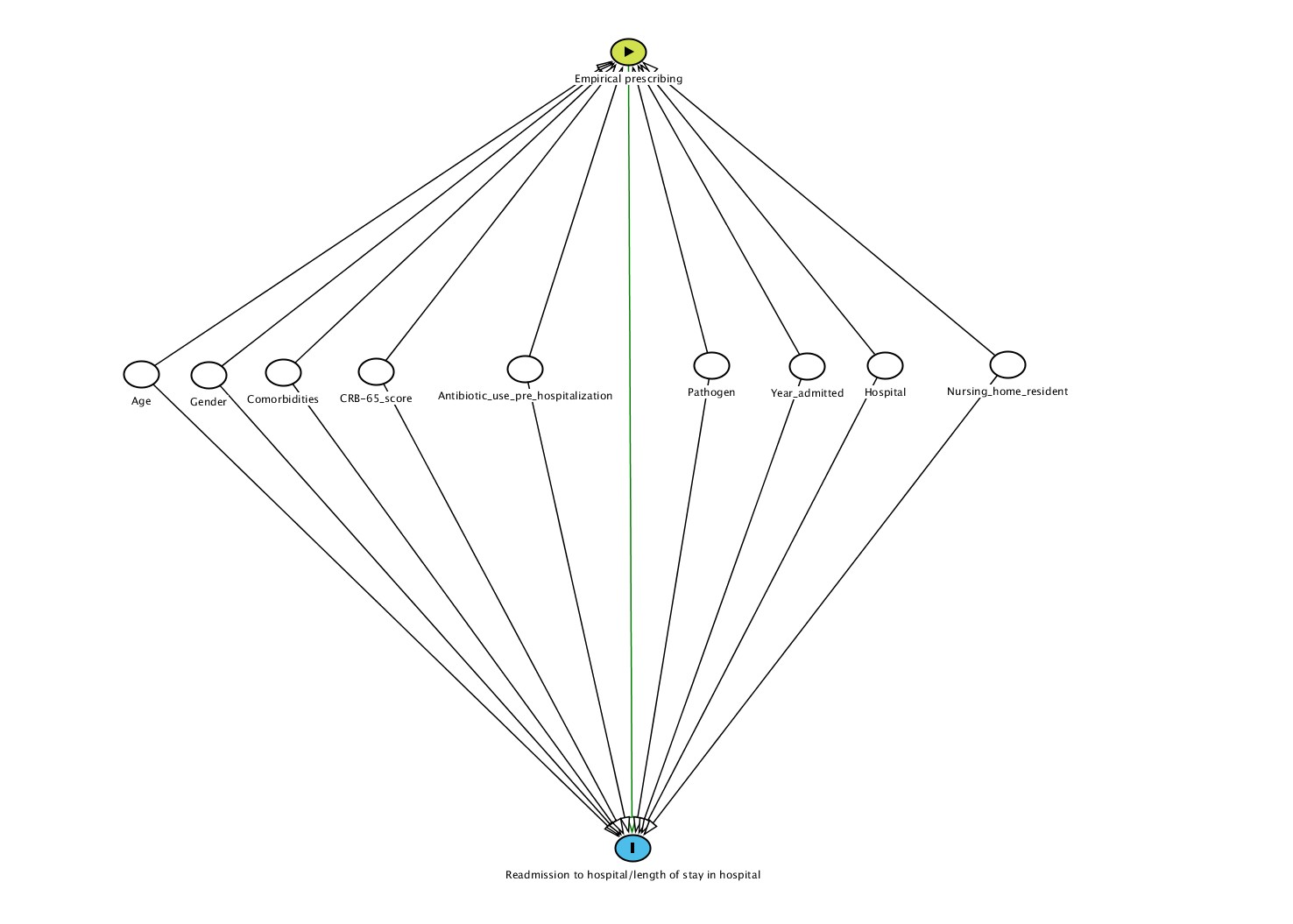

Supplement: Supplementary file 1 — Additional file 1. Directed Acyclic Graphs; Association between empirical prescribing (exposure) and readmission or length of stay in hospital (outcomes). [file 12890_2020_1188_MOESM1_ESM.docx]
