# Supplementary material for: Empirical prescribing of penicillin G/V reduces risk of readmission of hospitalized patients with community-acquired pneumonia in Norway: a retrospective observational study
Source: BMC Pulm Med. 2020 Jun 15;20:169. doi: 10.1186/s12890-020-01188-6 (PMC7294665; doi:10.1186/s12890-020-01188-6)
Supplement: Supplementary file 2 — Additional file 2. Description of all empirical antibiotics prescribed [file 12890_2020_1188_MOESM2_ESM.docx]

Additional file 2: Description of all empirical antibiotics prescribed (n=651)

| Empirical antibiotics prescribed | | n (%) | |
| --- | --- | --- | --- |
| Penicillin G/V | | 335 | (51.5) |
| Penicillin G + gentamicin | | 149 | (22.9) |
| Cephalosporines | | 83 | (12.7) |
| Erythromycin | | 22 | (3.4) |
| Amoxicillin/ampicillin | | 16 | (2.5) |
| Doxycycline | | 19 | (2.9) |
| Combination therapy | |  |  |
|  | Erythromycin + Penicillin G and/or gentamicin and/or cephalosporines | 6 | (1) |
|  | Cephalosporin + gentamicin | 6 | (1) |
|  | Diverse other combinations | 6 | (1) |
| Other | | 9 | (1.4) |
