# Supplementary material for: Empirical prescribing of penicillin G/V reduces risk of readmission of hospitalized patients with community-acquired pneumonia in Norway: a retrospective observational study
Source: BMC Pulm Med. 2020 Jun 15;20:169. doi: 10.1186/s12890-020-01188-6 (PMC7294665; doi:10.1186/s12890-020-01188-6)
Supplement: Supplementary file 3 — Additional file 3. Description of empirical antibiotic prescribed versus subsequent bacterial pathogen identified [file 12890_2020_1188_MOESM3_ESM.docx]

|  | Pathogen identified | | | | | |
| --- | --- | --- | --- | --- | --- | --- |
| Empirical  antibiotic choice | *S.pneumoniae* | *H.influenzae* | *M. or C.pneumonia* | *S.aureus* | *M.catarrhalis* | *Other* |
| Penicillin G | 20 | 4 | 10 | 6 | 2 | 6 |
| Penicillin G and gentamicin | 27 | 1 | 3 | 0 | 2 | 4 |
| Erythromycin | 0 | 2 | 4 | 0 | 0 | 0 |
| Cephalosporines | 8 | 2 | 0 | 1 | 0 | 0 |
| Other antibiotic combinations* | 5^a^ | 1^b^ | 2^c^ | 0 | 0 | 0 |
| Other | 1 | 0 | 2 | 0 | 0 | 0 |
| Doxycycline | 0 | 1 | 1 | 0 | 0 | 0 |
|  | | | | | | |
